# Recent Advances in Translational Pharmacokinetics and Pharmacodynamics Prediction of Therapeutic Antibodies Using Modeling and Simulation

**DOI:** 10.3390/ph15050508

**Published:** 2022-04-22

**Authors:** Kenta Haraya, Haruka Tsutsui, Yasunori Komori, Tatsuhiko Tachibana

**Affiliations:** 1 Discovery Biologics Department, Research Division, Chugai Pharmaceutical Co., Ltd., 1-135 Komakado, Gotemba 412-8513, Japan; tsutsui.haruka85@chugai-pharm.co.jp; 2 Pharmaceutical Science Department, Translational Research Division, Chugai Pharmaceutical Co., Ltd., 1-135 Komakado, Gotemba 412-8513, Japan; komori.yasunori40@chugai-pharm.co.jp (Y.K.); tachibanatth@chugai-pharm.co.jp (T.T.)

**Keywords:** therapeutic monoclonal antibodies, pharmacokinetics, pharmacodynamics, modeling and simulation, human prediction, TMDD, PKPD, PBPK, QSP, ADC

## Abstract

Therapeutic monoclonal antibodies (mAbs) have been a promising therapeutic approach for several diseases and a wide variety of mAbs are being evaluated in clinical trials. To accelerate clinical development and improve the probability of success, pharmacokinetics and pharmacodynamics (PKPD) in humans must be predicted before clinical trials can begin. Traditionally, empirical-approach-based PKPD prediction has been applied for a long time. Recently, modeling and simulation (M&S) methods have also become valuable for quantitatively predicting PKPD in humans. Although several models (e.g., the compartment model, Michaelis–Menten model, target-mediated drug disposition model, and physiologically based pharmacokinetic model) have been established and used to predict the PKPD of mAbs in humans, more complex mechanistic models, such as the quantitative systemics pharmacology model, have been recently developed. This review summarizes the recent advances and future direction of M&S-based approaches to the quantitative prediction of human PKPD for mAbs.

## 1. Introduction

Since the first therapeutic antibody, the anti-CD3 monoclonal antibody OKT3, was approved by the FDA in the 1980s, the development of therapeutic monoclonal antibodies (mAbs) has increased dramatically, especially over the last 10 years. Due to several advantages of mAbs against other therapeutic modalities, such as their strong and selective binding to the target antigen and their long half-life, mAbs have become major prescription drugs for several diseases. Moreover, antibody engineering has been used to expand upon the capabilities of conventional mAbs with the development of bispecific antibody (BsAb) [1], antibody drug conjugate (ADC) [2], recycling/sweeping antibody [3,4,5], and local tissue activable antibody [6,7,8]. MAbs will continue to be a major therapeutic option in the future.

The molecular weight of most mAbs is approximately 150 kDa due to their immunoglobulin G (IgG) structure. Cell penetration and glomerular filtration are limited due to this large molecular weight, leading to long half-life in blood after injection [9]. Moreover, mAbs are reported to show a longer half-life than other proteins with similar or higher molecular weights [10]. This is because mAbs are rescued from lysosomal degradation by neonatal Fc receptor (FcRn)-mediated recycling system [11]. The Fc region can only bind to FcRn at acidic pH. Thus, mAbs can hardly bind to cell surface FcRn in blood due to its neutral pH. After pinocytosis, mAbs move to the early endosome with pH around 6.0, then bind to FcRn via the Fc region. After forming complexes with FcRn, mAbs are recycled back to the cell surface together with FcRn from the endosome. Finally, they disassociate from FcRn and are released into the blood due to the neutral pH. This FcRn-mediated recycling system gives mAbs a long half-life in blood, around 5–30 days in humans [12]. To improve the efficiency of FcRn-mediated recycling and extend the half-life even further, several amino acid mutations in the Fc region to increase FcRn-binding affinity at acidic pH have been evaluated in animals and humans [13,14,15]. Among these mutations, M428L/N434S (LS) and M252Y/S254T/T256E (YTE) have been extensively investigated in several mAbs [5]. While conventional mAbs have a half-life of around 5–30 days in humans, mAbs with LS or YTE mutations showed a half-life of 50–80 days [16,17,18]. Other than modulating FcRn binding, reducing the isoelectric point (pI) or charge has also been shown to extend the half-life of mAbs [19]. Since the cell surface is negatively charged, mAbs with a lower pI or negative charge should be repulsed from the cell surface, slowing down pinocytosis and extending the half-life. Modulating pharmacokinetics of mAbs has been continually advanced by several engineering technologies. As with FcRn, the effect of Fc gamma receptors (FcγRs) binding on the pharmacokinetics of mAbs has been explored. Leabman et al. explored the effect of FcγR binding on the pharmacokinetics of mAbs in cynomolgus monkeys [20]. In this study, both decreased FcγR binding by mutagenesis and increased FcγR binding by afucosylation showed no influence on the pharmacokinetics. Zelevsky et al. also investigated the effect of increased FcγR binding in cynomolgus monkeys. This study showed that a 60-fold increase in FcγRIIIa binding did not have an influence on the pharmacokinetics of mAbs. Since increased FcγR binding can be an important approach to enhance the efficacy of mAbs, further investigation is required to conclude the effect of FcγR binding on the pharmacokinetics. As described above, mAbs have several unique characteristics beyond other therapeutic modalities. Although each characteristic has been mechanistically investigated by several groups, further quantitative and systemic analysis is needed for accelerating the development of mAbs.

Modeling and simulation (M&S) is applied in a variety of ways in drug discovery and development. Among these, the prediction of human PKPD ahead of clinical trials is an application that can greatly impact the success and efficiency of clinical development. In the general drug discovery and development process, several in vivo animal studies and in vitro studies are conducted to select clinical candidates. Then, before starting clinical trials, the success rate of the selected clinical candidate must be predicted based on in vivo animal data, in vitro data, and published clinical data. Although such data supports the assessment of clinical candidates, quantitative decision making could be difficult without M&S. M&S can quantitatively combine several types of data and extrapolate in humans, enabling quantitative decision making of the discovery and development of mAbs.

M&S essentially requires optimal models that can be used to analyze and simulate a variety of profiles, such as PKPD. Traditionally, the compartment model has been used to capture the pharmacokinetics of mAbs, and E_max_-based models such as the direct/indirect response model and tumor growth inhibition model have been used for pharmacodynamics. These models are highly useful since they can be applied to a wide variety of data. However, detailed PKPD mechanisms are not incorporated into these models. Thus, mechanism-based models are highly required to analyze and translate preclinical in vivo and in vitro data to humans more accurately. Recently, new mechanistic models such as the target-mediated drug disposition (TMDD) model, physiologically based pharmacokinetic (PBPK) model, and quantitative systems pharmacology (QSP) model have been applied to mAbs. These models can be used to translate preclinical in vivo/in vitro PKPD to humans. In this review, we summarize how M&S can be used to quantitatively predict human PKPD for therapeutic mAbs and discuss future directions.

## 2. Traditional Model-Based Prediction of Human PKPD

### 2.1. Compartment-Model-Based Prediction of Linear Pharmacokinetics

**Figure 1:**
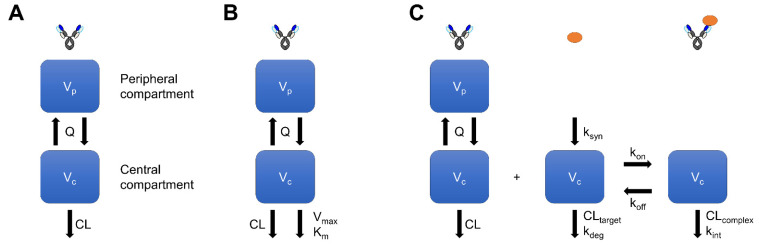
Basic model structure of (**A**). two-compartment model, (**B**). Michaelis–Menten model, (**C**). target-mediated drug disposition model. CL; clearance, Q; inter-compartmental CL, V_c_; volume of distribution in the central compartment, V_p_; volume of distribution in the peripheral compartment, K_m_; Michaelis constant, V_max_; maximum rate of nonlinear elimination, k_syn_; synthesis rate of target antigen, CL_target_/k_deg_; elimination clearance/rate constant of target antigen, CL_complex_/k_int_; elimination clearance/internalization rate constant of complex, k_on_; association rate constant, k_off_; dissociation rate constant.

The pharmacokinetic profile of mAbs is largely classified into two types: linear and nonlinear. Generally, linear pharmacokinetics is governed mainly by nonspecific pinocytosis and the FcRn-mediated recycling system. Since both processes have huge and unsaturated capacity at realistic dosage, mAbs with linear pharmacokinetics such as bevacizumab (approved anti-VEGF antibody) [21], adalimumab (approved anti-TNFα antibody) [22], and mepolizumab (approved anti-IL-5 antibody) [23] show dose-dependent exposure and a constant half-life in a dose-independent manner. Pharmacokinetic profiles of mAbs with linear pharmacokinetics after intravenous injection have been reported to be biphasic (distribution phase and elimination phase) [24,25]. Therefore, a two-compartment model (Figure 1A) has been frequently used to capture the plasma mAbs concentration–time profile after intravenous injection in both humans and animals [26,27]. Generally, the two-compartment model uses only four pharmacokinetic parameters (CL, Q, V_c_, and V_p_, or CL, V_c_, k_12_, and k_21_). Since the elimination process directly affects CL, a wide variety of CL values have been shown for several mAbs [28,29,30]. On the other hand, the tissue distribution process affects Q, V_c_, V_p_, k_12_, and k_21_. Since the tissue distribution of mAbs is limited due to their large molecular weight, a similar Q, V_c_, and V_p_ has been reported for several mAbs [29] in cynomolgus monkeys and humans [30]. The prediction of four pharmacokinetic parameters can be used to predict the whole-plasma mAbs concentration–time profile after intravenous injection in humans. This method can quantitatively contribute to the decision of a project and design of a clinical trial.

Several animals (mice [31], rats [32], rabbits [33], dogs [34], pigs [35], and monkeys [36]) have been used to preclinically evaluate the pharmacokinetics of mAbs in vivo. Among these, cynomolgus monkeys are the most widely used to predict the pharmacokinetics of mAbs in humans because the two species have similar FcRn-binding affinity against mAbs [37,38]. Several studies have shown that the clearance and volume of distribution of mAbs in humans can be accurately predicted from that in cynomolgus monkeys using the allometric scaling approach [39,40]. Deng et al. demonstrated that single species allometric scaling using cynomolgus monkeys showed better prediction accuracy of human pharmacokinetics than multiple-species allometric scaling [40]. This would be due to inter-species difference of FcRn binding between rodents and humans. Although several case studies, such as BIIB059 (anti-BDCA2 antibody) [41], M701 (anti-CD3/EpCAM bispecific antibody) [42], and JNJ-61178104 (anti-TNFα/IL-17A bispecific antibody) [43], have used the allometric scaling approach with cynomolgus monkeys to predict two-compartment model parameters in humans, each of these case studies used a different scaling exponent without solid evidence for an optimal exponent. Thus, we established an optimal exponent for the allometric scaling of all two-compartment model parameters of mAbs in humans based on cynomolgus monkey data. We achieved this by comprehensively analyzing pharmacokinetic data on 24 mAbs in cynomolgus monkeys and humans [44]. The optimal exponents for CL, Q, V_c_, and V_p_ were 0.8, 0.75, 1.0, and 0.95, respectively. Using cynomolgus monkey data, our approach accurately predicted the whole-plasma mAbs concentration–time profile after intravenous injection in humans. Furthermore, by combining parameters predicted using the two-compartment model and subcutaneous absorption parameters (bioavailability (F) and absorption rate constant (k_a_)), we were also able to accurately predict the plasma mAbs concentration–time profile after subcutaneous injection in humans. In this approach, subcutaneous F was predicted based on clearance in humans, and k_a_ was assumed to be a geometric mean of reported values for several mAbs. The two-compartment-model-based prediction approach of human mAbs pharmacokinetics from cynomolgus monkeys was successfully established using a validated optimal exponent. Another compartment model approach to predict the plasma mAbs concentration–time profile after subcutaneous injection in humans has also been reported [45]. Since the plasma mAbs concentration–time profile after subcutaneous injection has been reported to be captured by a one-compartment model with a first-order absorption and elimination process, only three parameters (apparent CL (CL/F), apparent volume of distribution (V_d_/F), and k_a_) are sufficient for prediction. This analysis established optimal exponents for scaling CL/F, V_d_/F, and k_a_ from cynomolgus monkeys to humans and accurately predicted the plasma mAbs concentration–time profile of 13 mAbs after subcutaneous injection in humans. This approach is simple and requires no intravenous injection data, thus improving animal welfare and reducing costs. Additionally, Shivva et al. investigated the inclusion of inter-individual variability for predicting human pharmacokinetics of mAbs from non-human primates [46]. The application of scaled half-life from non-human primates as covariates on parameters accurately predicted the inter-individual variability of parameters in humans. This approach would contribute to more the efficient design of a first-in-human study.

Although the cynomolgus monkey is known to be a useful animal for predicting the pharmacokinetics of mAbs in humans, it is very expensive and evaluation requires a large amount of mAbs. Recently, human FcRn transgenic mice (hFcRn Tgm), which express human FcRn as a substitute for mouse FcRn, have been proposed as an alternative to cynomolgus monkeys for the pharmacokinetic screening of mAbs and the prediction of clearance in humans [47,48]. Generally, mice are a useful animal for in vivo screening candidate molecules because they are easy to handle, require only small amounts of molecules for evaluation, and are better in terms of animal welfare [49]. However, inter-species differences in FcRn binding have been reported between mice and humans [50]. In a surface plasmon resonance (SPR) assay, mouse FcRn showed much stronger binding activity to human IgG compared with human FcRn. Moreover, due to the poor correlation in the pharmacokinetics of the two species, mice are inadequate for human prediction [47]. As previously mentioned, due to inter-species similarities in FcRn binding, cynomolgus monkeys are normally used to predict mAbs pharmacokinetics in humans; however, hFcRn Tgm may also be able to overcome the inter-species difference in FcRn binding. Betts et al. investigated the use of hFcRn Tgm to predict the plasma mAbs concentration–time profile in humans [51]. This study demonstrated comparable prediction accuracy of two-compartment model parameters in humans by allometric scaling from cynomolgus monkeys or hFcRn Tgm. Although hFcRn Tgm cannot be used to evaluate the effect of target antigen binding on mAbs pharmacokinetics, in many cases, due to no or weak cross-reactivity to the mouse target antigen, it would be useful for evaluating and predicting human linear pharmacokinetic parameters of mAbs.

The two-compartment-model-based approach to predict human pharmacokinetics has already been validated by several studies [44,51]. However, it usually requires preclinical in vivo data from animals such as cynomolgus monkeys and hFcRn Tgm. An in vitro or in silico-based prediction method would be more affordable, better for animal welfare, and would speed up drug development. Since several mAbs have shown similar Q, V_c_, V_p_ among several mAbs in humans, prediction of only CL by an in vitro/in silico approach would be sufficient to predict the whole-plasma mAbs concentration–time profile in humans by fixing Q, V_c_, and V_p_. Additionally, the applicability of the current approach to new engineered mAbs needs to be validated for each case. Since several molecular formats have been developed, applicability should be carefully checked.

### 2.2. Michaelis–Menten-Model-Based Prediction of Nonlinear Pharmacokinetics

Nonlinear pharmacokinetics of mAbs has been frequently observed, especially when affected by target antigen expression. Denosumab is an anti-RANKL monoclonal antibody that showed nonlinear pharmacokinetics in humans [52]. Slower clearance observed at the higher dose of denosumab would be due to the saturation of target-antigen-mediated clearance. Nonlinear pharmacokinetics has been observed in humans for several mAbs, i.e., cetuximab (anti-EGFR antibody) [53], volociximab (anti-α5β1 antibody) [54], lumiliximab (anti-CD23 antibody) [55], elotuzumab (anti-SLAMF7 antibody) [56], dalotuzumab (anti-IGF-1R antibody) [57], TRC105 (anti-endoglin antibody) [58], icrucumab (anti-VEGFR-1 antibody) [59], BIIB023 (anti-TWEAK antibody) [60], EMD 525797 (anti-αv-integrin antibody) [61], and brodalumab (anti-IL-17R antibody) [62]. Nonlinear pharmacokinetics of mAbs in humans was traditionally predicted from cynomolgus monkeys using the species time-invariant method [63]. Although this approach is useful to simply translate the pharmacokinetic profile of cynomolgus monkeys to humans, the predicted pharmacokinetics profile cannot be modified according to study design because it does not use a mathematical model. Therefore, the human pharmacokinetic profile predicted using the species time-invariant method was re-analyzed by the model; then, the pharmacokinetic profiles for several situations were simulated in this approach.

To quantitively analyze the nonlinear pharmacokinetics of mAbs, Michaelis–Menten (MM) (Figure 1B) and target-mediated drug disposition (TMDD) (Figure 1C) models have been frequently used. The MM model generally uses the Michaelis constant (K_m_) and the maximum rate of nonlinear elimination (V_max_) as nonlinear pharmacokinetic parameters. Due to the simple structure of the equation without mechanistic parameters, the MM model can be applied to a wide variety of situations even if the mechanism of nonlinear pharmacokinetics or target antigen information is unknown. On the other hand, even if there is an inter-species difference in mechanistic parameters, such as the turnover of the target antigen or binding affinity between human and cynomolgus monkey, prediction cannot be quantitatively adjusted by such factors in the MM model. Dong et al. systemically investigated the prediction accuracy of human nonlinear pharmacokinetics of mAbs from cynomolgus monkeys by the MM model using in total six mAbs [64]. Linear pharmacokinetic parameters in humans were predicted using the allometric scaling approach with a fixed exponent. K_m_ in humans was assumed to be the same in cynomolgus monkeys and V_max_ in humans was allometrically scaled with an exponent of 0.75 from that in cynomolgus monkeys. Although plasma mAbs concentration–time profiles in humans at high concentrations were reasonably predicted, those at low concentrations were poorly predicted, suggesting the need to further improve the scaling methodology. The MM-model-based approach has been applied to several mAbs such as E6011 (anti-CX3CL1 antibody) [65], MCLA-128 (anti-HER2/HER3 bispecific antibody) [66], and SI-B001 (anti-EGFR/HER3 bispecific antibody) [67]. Recently, Singh et al. investigated the utility of three approaches (species time-invariant method, MM model, and minimal physiologically based pharmacokinetics (mPBPK) model) to predict the nonlinear pharmacokinetics of mAbs in humans from cynomolgus monkey data using five mAbs [68]. As a result of the comparison analysis, the species time-invariant method and mPBPK model showed better prediction accuracy for nonlinear pharmacokinetics in humans compared with the MM model. According to several reports, although the MM model works for some situations, mechanistic models would be more accurate for predicting nonlinear mAb pharmacokinetics in humans.

### 2.3. Traditional Model-Based Prediction of Pharmacodynamics

Pharmacodynamics is one of the most important outputs to determine the value of therapeutics. Therefore, prediction of pharmacodynamics in humans before starting clinical trials can be crucial for the success of the selected clinical candidate. Traditionally, pharmacodynamics was directly predicted from animal study data under the assumption that the efficacious plasma mAbs concentration between humans and animals was the same. Xiang et al. aimed to predict the clinical target dose of onartuzumab (anti-Met antibody) from animal data [69]. First, they conducted a xenograft mouse efficacy study with a human tumor cell line and estimated the tumoristatic concentration (TSC) of onartuzumab using a tumor growth inhibition model. Then, the clinical efficacious dose was predicted to maintain plasma trough concentration at a steady state above TSC. This approach assumes a comparable exposure–anti-tumor activity relationship between the humans and xenograft mouse model. A similar tumor-growth-inhibition-model-based approach has been reported for PRO95780 (anti-DR5 antibody) [70], rhuMAb VEGF (anti-VEGF antibody) [71], SI-B001 (anti-EGFR/HER3 bispecific antibody) [67], and MCLA-128 (anti-HER2/HER3 bispecific antibody). Since the mechanistic process is not incorporated into this model, it cannot account for the mechanism-based inter-species differences in effective concentration. Thus, selection of tumor cell lines and mouse models, such as the huNOG mouse model and human PBMC injection mouse model, can be an important factor for the prediction accuracy of this approach.

Traditional model-based pharmacodynamics prediction has been also conducted for the treatment of diseases other than cancer. The pharmacodynamics of the anti-BDCA2 antibody BIIB059, a treatment for systemic lupus erythematosus (SLE), was predicted in humans from preclinical data [41]. An indirect response pharmacodynamics model was used to capture the free BDCA2 level on plasmacytoid dendric cells (pDC) after BIIB059 injection in cynomolgus monkeys. Then, EC50 was scaled from cynomolgus monkeys to humans by considering the species difference in the number of pDC and BDCA2 expression level on pDC. Other parameters were assumed to be the same in the two species. This approach successfully predicted the observed pharmacodynamics of BIIB059 in a phase 1 study. Indirect response pharmacodynamics model-based human prediction using cynomolgus monkeys was also reported for the anti-FcRn antibody rozanolixizumab [72]. Additionally, the clinically observed pharmacodynamics (muscle increment) of the anti-myostatin antibody MYO-029 was interpretated using a direct response model [73]. MYO-029 showed insufficient efficacy in clinical trials. Therefore, the exposure–response relationship in mice, monkeys, and humans was analyzed using the direct response model. Due to similarity in myostatin expression and muscle physiology, the exposure–response relationship in monkeys was selected for predicting human efficacy. As a result, if the exposure–response relationship in humans was assumed to be same in monkeys, insufficient myostatin coverage of MYO-029 in clinical trials was shown. Translational human PKPD prediction can be used to avoid this kind of case and improve the probability of success in clinic. These traditional model-based predictions could be useful when the target antigen biology is unclear or comparable in animals and humans. However, if mechanistic translation from animals to humans is required for target antigen biology, a mechanism-based model should be constructed and applied for human PKPD prediction.

## 3. TMDD-Model-Based Prediction of Human PKPD

### 3.1. TMDD-Model-Based Prediction of Nonlinear Pharmacokinetics

As described above, nonlinear pharmacokinetics of mAbs has been frequently described by the MM or TMDD model. The TMDD model generally incorporates target antigen binding parameters (equilibrium dissociation constant (KD), association rate constant (k_on_), dissociation rate constant (k_off_), etc.) and target antigen parameters (total target antigen concentration (R_tot_), synthesis rate of target antigen (k_syn_), and elimination/internalization rate constant (k_deg_/k_int_)). The TMDD model can incorporate more mechanistic processes than the MM model. Therefore, the mechanism-based prediction of human pharmacokinetics can be conducted using the TMDD model by incorporating target antigen information in humans. The basic TMDD-model-based strategy is to scale the nonlinear pharmacokinetics in cynomolgus monkeys to humans. Luu et al. have used the TMDD model to predict the human pharmacokinetics of the anti-ALK1 monoclonal antibody PF-03446962 [74]. First, they conducted a pharmacokinetic study in cynomolgus monkeys at several doses and observed the nonlinear pharmacokinetics. Then, they analyzed the nonlinear pharmacokinetics using the TMDD model and estimated each parameter. To translate parameters from cynomolgus monkeys to humans, linear pharmacokinetic parameters were scaled by the allometric scaling approach. Moreover, they estimated the target antigen binding parameters and internalization rate constant of the PF-03446962-ALK complex (k_int_) in humans from in vitro studies, although the target antigen expression was assumed to be the same in cynomolgus monkeys and humans. Additionally, they estimated the elimination rate constant of ALK1 (k_deg_) based on the reported in vitro half-life of human ALK1. Finally, their approach accurately predicted the human pharmacokinetics of PF-03446962 as observed in the clinical trial. A similar approach has been applied to several mAbs, such as PF-06741086 (anti-TFPI antibody) [75], GC1118 (anti-EGFR antibody) [76], Tovetumab (anti-PDGFRα antibody) [77], MG1113 (anti-TFPI antibody) [78], PF-04840082 (anti-Dkk-1 antibody) [79], AMG 181 (anti-α4β7 antibody) [80], TAM-163 (anti-TrkB antibody) [81], and QBP359 (anti-CCL21 antibody) [82].

Although several groups have reported case studies in which the TMDD model was used to predict nonlinear pharmacokinetics of mAbs in humans as shown above, a comprehensive analysis of TMDD-model-based human prediction was needed to judge its utility. Singh et al. were the first to conduct such an analysis using seven mAbs in non-human primates that included cynomolgus monkeys, chimpanzees, and baboons [83]. First, using six mAbs, they conducted a correlation analysis of each TMDD model parameter in humans and non-human primates to develop translational rules. Two compartment model parameters in humans can be scaled from non-human primates using the allometric scaling approach or assumed to be the same with non-human primates. On the other hand, target antigen binding parameters and target antigen turnover parameters in humans can be assumed to be the same in non-human primates or can be adjusted based on in vitro experimental data. Using these translational rules, they predicted the nonlinear pharmacokinetics of one test antibody in humans from cynomolgus monkey data. Most of the data for the test antibody was well predicted, demonstrating the validity of this approach.

In most cases targeting membrane-expressed target antigen, the target antigen expression level has been assumed to be comparable in animals and humans. However, the difference in expression level between animals and humans has been reported to be highly dependent on the protein [84,85]. Thus, if the expression level of a target antigen is different in animals and humans, the difference must be included in the model for the prediction to be accurate. Ahlberg et al. investigated the applicability of mRNA expression data to extrapolate the preclinical nonlinear pharmacokinetics to humans [86]. Their anti-IL-36R monoclonal antibody, MAB92, showed human IL-36R specific binding (over 2000-fold weaker binding affinity against rodent and nonhuman primate IL-36R). Thus, the effect of IL-36R on pharmacokinetics of MAB92 could not be evaluated in animals. To evaluate the effect of IL-36R on the pharmacokinetics of the anti-IL-36R antibody in animals, the surrogate anti-mouse IL-36R monoclonal antibody MAB04 was generated and tested in mice. MAB04 showed nonlinear pharmacokinetics in mice and observed pharmacokinetics was analyzed by the TMDD model. To predict the nonlinear pharmacokinetics of MAB92 in humans, first, linear pharmacokinetic parameters were extrapolated from those in cynomolgus monkeys using allometric scaling, and nonlinear pharmacokinetic parameters were assumed to be the same as those in mice, although the binding affinity was verified by in vitro data. This first approach assumed that IL-36R expression in humans was the same in mice, resulting in the clear underprediction of AUC in humans. Thus, secondly, IL-36R expression was adjusted based on the difference in mRNA expression between mice and humans. As a result, the nonlinear pharmacokinetics of MAB92 in humans was accurately predicted across several doses. This study demonstrated that target antigen expression data is needed to accurately predict nonlinear pharmacokinetics in humans. Although mRNA expression data was used in this study, mRNA expression has been reported to be poorly correlated with protein expression [87]. Thus, incorporating protein expression data would be more effective for obtaining the accurate target antigen expression level.

The structure of the MM and TMDD models were established based on several past studies [88,89]. Moreover, the predictability of nonlinear pharmacokinetics in humans using these models has been repeatedly demonstrated [64,74]. Although the MM model seems not to be further customizable, there is still room to improve the methodology of the TMDD-model-based approach. Currently, TMDD model parameters are often scaled from cynomolgus monkeys. However, if an optimal in vitro assay system were established to estimate target antigen turnover in humans, the inter-species difference in turnover could be incorporated to achieve more accurate human prediction.

### 3.2. TMDD-Model-Based Prediction of Pharmacodynamics

The TMDD model has been used to predict not only nonlinear pharmacokinetics but also target antigen occupancy in humans as pharmacodynamics. There are two main types of target antigen: soluble target antigens such as cytokines, chemokines, and toxins, and membrane-bound target antigens such as receptors, transporters, and channels. Soluble target antigen is secreted from specific cells into extracellular space and maintains a certain endogenous concentration in the body. Since the plasma-soluble target antigen concentration can be easily quantified by conventional bioanalytic technologies, it has been used to mathematically determine target antigen occupancy by mAbs using target antigen concentration, mAbs concentration, and affinity. Although target antigen occupancy can be calculated with a simple equation, the TMDD model can simulate the dynamics of target antigen occupancy under several conditions by incorporating mechanistic parameters.

Dudal et al. investigated the use of the TMDD model to predict the target antigen occupancy of the anti-CCL21 antibody QBP359 in humans from cynomolgus monkeys [82]. CCL21 is a soluble chemokine and would be involved in inflammation-related conditions. First, QBP359 was evaluated in cynomolgus monkeys. The pharmacokinetics of QBP359 and the total plasma concentration of CCL21 after the QBP359 injection were evaluated and analyzed using the TMDD model. Estimated parameters in cynomolgus monkeys were allometrically scaled to humans. Moreover, since plasma CCL21 concentration in humans is reported to be 10-fold higher than that in cynomolgus monkeys, this difference was incorporated into the TMDD model. Using the estimated parameters, CCL21 occupancy in humans was predicted for several dosing regimens of QBP359. The predicted result showed that high and frequent dosing of QBP359 in clinical situations would be required to sufficiently suppress CCL21 in tissues, suggesting that QBP359 would be difficult to develop clinically. This analysis can support decision making when selecting candidate molecules in the preclinical stage. Similarly, the TMDD model has been used to predict target antigen occupancy of soluble target antigen as pharmacodynamics in humans from cynomolgus monkeys using in several mAbs, such as PF-04840082 (anti-Dkk1 antibody) [79], JNJ-61178104 (anti-TNFα/IL-17A bispecific antibody) [43], PF-06741086 (anti-TFPI antibody) [75], MG1113 (anti-TFPI antibody) [78], and E6011 (anti-CX3CL1 antibody) [65].

Membrane-bound target antigen is another target for mAbs. Since membrane-bound target antigen is expressed on cell membranes, it can be challenging to quantify the absolute value of expression in the body using conventional analysis. Therefore, TMDD-model-based estimation of target antigen occupancy by mAbs in animals has been conducted to predict pharmacodynamics in humans. Park et al. investigated the prediction of target antigen occupancy by the anti-EGFR antibody GC1118 in humans from cynomolgus monkeys using the TMDD model [76]. EGFR is a cell membrane growth factor receptor and is reported to be overexpressed in several types of tumor. Due to EGFR-mediated clearance, GC1118 showed nonlinear pharmacokinetics in cynomolgus monkeys. The nonlinear pharmacokinetics of GC1118 in cynomolgus monkeys was analyzed by the TMDD model and translated to humans. In this analysis, two-compartment model parameters (clearance of EGFR, clearance of EGFR-GC1118 complex, and the synthesis rate of EGFR) were scaled from cynomolgus monkeys to humans. In vitro data was used to estimate the affinity of GC1118 to EGFR. Using the estimated parameters, the nonlinear pharmacokinetics of GC1118 and EGFR occupancy in humans was predicted, which contributed to the design of the GC1118 dosing regimen in the clinical trial. The prediction of occupancy for membrane-bound target antigen in humans from cynomolgus monkeys using the TMDD model was also reported for TAM-163 (anti-TrkB antibody) [81], Tovetumab (anti-PDGFRα antibody) [77], and AMG 181 (anti-α4β7 antibody) [80]. The predicted occupancy in humans can be used to judge the clinical potential of selected candidate molecules and design the dosing regimen for clinical trials. Although the conventional TMDD model can accurately describe target-antigen-mediated kinetics, it does not express TMDD in the tissue compartment. Thus, the combination of the TMDD and PBPK model would be more effective at capturing target-antigen-mediated kinetics in tissue. This concept will be described in the next section.

As shown in Figure 1C, the mAb–target antigen binding process utilizes k_on_ and k_off_ in the TMDD model. However, due to multiple parameters in the TMDD model and an insufficient pharmacokinetic data set, each parameter would not be identifiable in several cases. Therefore, a simpler TMDD model called the quasi-equilibrium (QE) model has been proposed [90]. The QE model assumes that mAb-target antigen association and dissociation are much faster than other processes. Thus, in this model, target antigen occupancy is assumed to be determined by only the free mAb concentration and KD (k_off_/k_on_). However, if other processes, especially clearance of mAb–target antigen complex, cannot be negligible against k_off_, the QE model would not be optimal. In such cases, the quasi-steady-state (QSS) model can be used to estimate target antigen occupancy. Gibiansky et al. investigated the difference in prediction from full TMDD, QE, QSS, and MM models [91]. When k_int_ was much smaller than k_off_, the QE model could sufficiently estimate parameters. On the other hand, when k_int_ was not negligible against k_off_, the QE model was not valid. Although most recent reports have used the full TMDD model for human prediction, its structure and the balance between k_int_ and k_off_ should be carefully checked.

## 4. PBPK-Model-Based Prediction of Human PKPD

PBPK models describe the pharmacokinetic profiles of mAbs based on mechanistic processes which incorporate measurable physiological and drug-specific parameters. PBPK-model-based PKPD modeling of mAbs is a useful tool for selecting candidates based on in vitro and in vivo parameters, and for the translational prediction of pharmacokinetics, including tissue distribution and the mode of action (MoA) in different animal species [92].

Due to their large molecular weight, mAbs show lower distribution into tissues compared with small molecule drugs. MAbs, especially those with topical targets, need to achieve considerable exposure at the target site. There are several quantitative methods for determining the tissue distribution of mAbs at the macro and micro scale. Traditionally, the whole tissue concentration of mAbs can be determined using ligand binding assay (LBA), liquid chromatography–mass spectrometry (LC–MS), and by measuring the radioactivity of radiolabeled mAb after preparing tissue homogenate from excited tissues [93]. Semi-quantitative immunohistochemistry (IHC) or quantitative whole-body autoradiography (QWBA) and fluorescent imaging are also tools that can measure the concentrations of mAbs in a whole tissue by preparing the cross-sections of tissues and the whole body [94,95,96]. These methods can detect an administered mAb in tissues at high sensitivities. However, the measured concentrations in whole tissues do not necessarily reflect the exact concentrations in the extracellular space where mAbs can distribute, because the distribution manner of mAbs in each sub-tissue compartment is not completely homogenous due to transport barriers [97]. In addition, the measured radioactivity might not completely reflect the concentrations of mAbs themselves. Tissue distribution data of mAbs labeled with iodine-125 (I-125) and indium-111 (In-111) enable us to identify the tissues where mAbs cumulatively distribute and degrade, because I-125 is rapidly released and eliminated from the system and In-111 is accumulated within cells due to its polarity or charge. However, signals from I-125 or In-111 after the administration of labeled mAbs with each isotope do not necessarily reflect the concentrations of intact mAbs or the sum of intact and degraded mAbs, respectively, because the half-lives of mAbs and each isotope are different [98]. These are also highly invasive methods which are difficult to apply in a clinical study. Positron emission tomography (PET) or single-photon emission computed tomography (SPECT) can enable the non-invasive and highly sensitive quantification of tissue concentrations, but these methods also provide low resolutive distribution data [99]. Micro-scale measurement methods such as micro-autoradiography (micro-ARG), matrix-assisted laser desorption/ionization mass spectrometry imaging (MALDI-MSI), and intravital microscopic imaging (IVM) are highly resolutive methods which can capture the mAb concentration in the tissue microenvironments in either an invasive or minimally invasive manner [100,101,102,103]. More specifically, microdialysis and tissue centrifugation methods can indirectly or directly isolate the tissue interstitial fluid to determine the mAb concentrations at the site of distribution [104,105].

### 4.1. Physiological Parameters and Model Structure in PBPK Model

**Figure 2:**
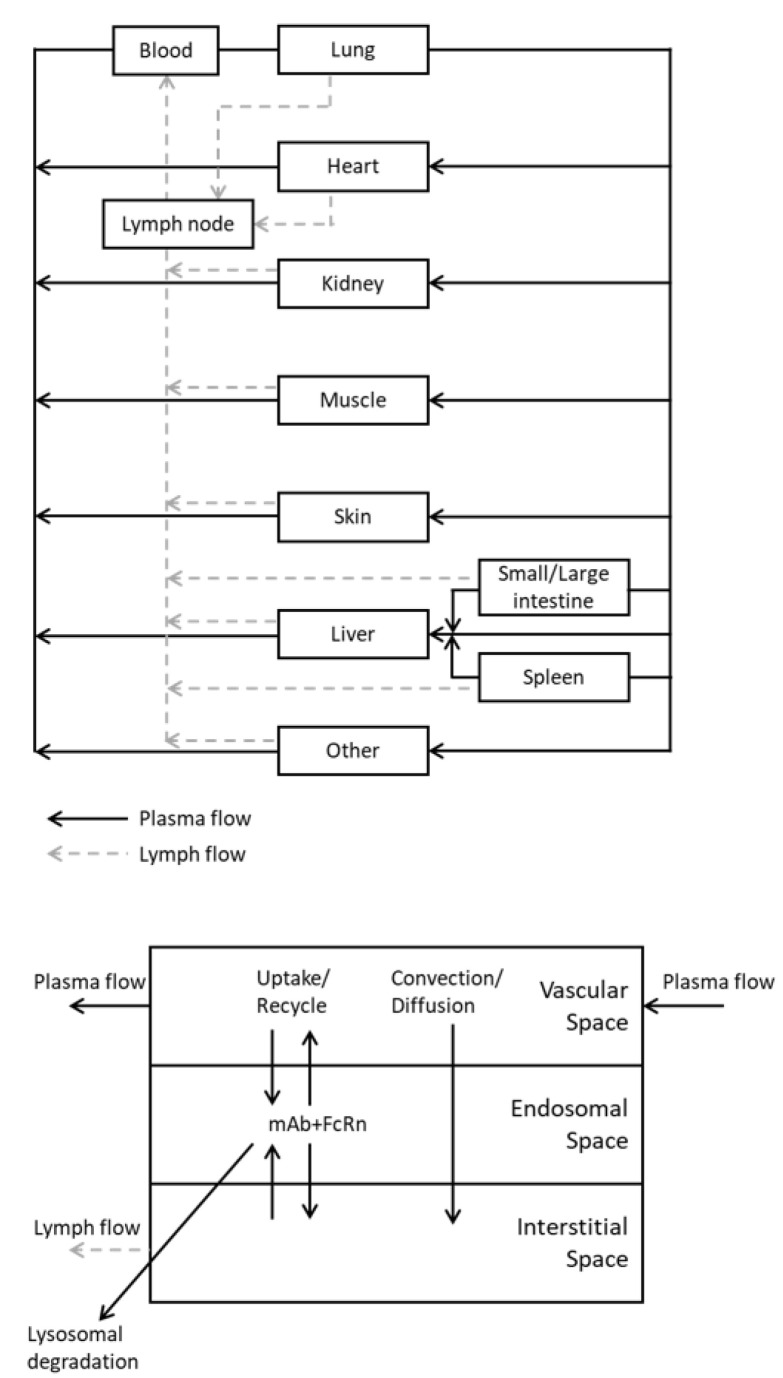
Schematic representation of the PBPK model.

Several PBPK models have been established by using the biodistribution data of mAbs [106,107,108,109]. The common structural features of these models are: the organs are connected via the blood and lymph flows and each organ is divided by several compartments to explain the typical pharmacokinetic profile of mAbs; tissue distribution via the convective and diffusive transport between the plasma and interstitial space across the endothelial cells; pinocytotic uptake and elimination via lysosomal degradation in the endothelial cells; and FcRn-mediated salvage from lysosomal degradation, as shown in Figure 2. To express the pharmacokinetics of mAbs, the extracellular space of organs—into which mAbs are distributed—are often divided into vascular, endothelial, and interstitial space. There are also some differences in the structures and/or physiological parameters of each model.

1. Plasma and lymph flow rate

**Figure 3:**
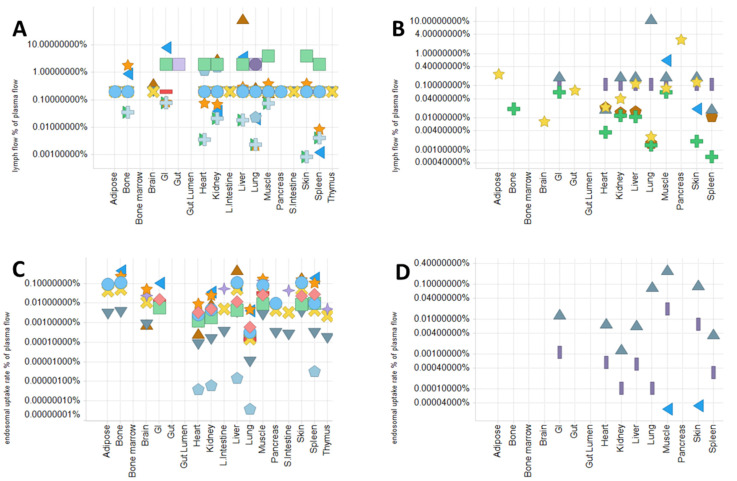

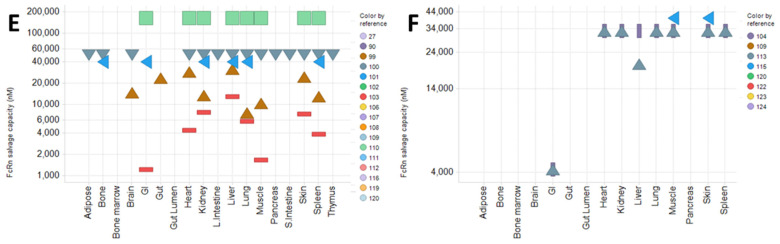
Representative physiological parameter values used in PBPK models in the different literature Literature values of percentage of lymph flow per plasma flow (**A**,**B**), percentage of endosomal uptake clearance per plasma flow (**C**,**D**), and FcRn concentration (**E**,**F**) of mouse (**A**,**C**,**E**) or human (**B**,**D**,**F**) in each organ used for PBPK model. Each symbol represents a different literature. The total endosomal volume in catenary model was calculated as five-fold volume of the endosomal sub-compartment [110,111,120].

The values of plasma flow rate used in multiple studies are almost comparable (at most a 10-fold difference) in both mice and humans. Some models assume the constant ratio (0.11 to 4%) of the lymph flow rate against the plasma flow rate in all tissues [31,107,110,111], but there is more than a 10-fold difference between studies in these ratio values. Moreover, some models estimate the specific lymph flow rate for each tissue, and these values differ depending on the study (Figure 3A,B).

2. Rate of pinocytosis, lysosomal degradation, and FcRn-related parameters

MAbs, as well as endogenous IgG, undergo pinocytosis into vascular endothelial cells, catabolic degradation in the lysosomes, and FcRn-mediated recycling into vascular and interstitial space. By incorporating parameters related to these processes into the PBPK model, we can capture and predict the effect of modification of FcRn-binding characteristics on the pharmacokinetics of mAbs. Many studies include the FcRn-mediated recycling mechanism in the PBPK model structure for mAbs [97,106,107,108,110,111,112,113,114,115,116,117,118,119,120,121]. However, those values differ greatly between studies which refer to the different reference data for calculating the affinity or expression of FcRn; pinocytosis rate, lysosomal degradation rate, FcRn concentration, and FcRn-mediated recycling rate range from 0.0002 to 13.8/day, 0.01 to 1030/day, 1.2 × 10^3^ to 1.6 × 10^5^ nM, and 5.0 to 68/day, respectively [33,106,107,108,110,111,113,114,115,116,117,118,119,120,121,122,123] (Figure 3C–F). Furthermore, the interaction between FcRn and mAbs in the endothelial endosomal space is assumed to occur by either equilibrium kinetics or catenary binding kinetics. Urva et al. and Garg and Balthasar depicted the endosomal space as a single compartment assuming the equilibrium-binding interaction of FcRn and mAbs [97,119]. Chen and Balthasar divided the endosomal space into five compartments based on pH conditions to describe the time-dependent endosomal transit of mAbs [110,120].

3. Recirculation flow rate (for the two-pore model) and lymphatic/vascular reflection coefficients

MAbs distribute into the interstitial space of each tissue via the convective and diffusive transcapillary transport across the endothelial membrane. For describing this transport process, there are two theories which assume either the homoporous or heteroporous endothelial membrane: one-pore and two-pore theories [31,109]. In the one-pore model, the net transport rate (*J_t_)* caused by the difference in hydrostatic and osmotic pressure across the capillary membrane is expressed in the following equation using the mAb concentrations in vascular and interstitial spaces expressed as *C_p_* and *C_i_* [31,124]:

(1) $$J_{t}=J_{v}\left(1-\sigma \right)\bar{C}+PS\left(C_{p}-C_{i}\right)$$

where $\bar{C}$ is the averaged intramembrane mAb concentration, which approximately equals (*C_p_* + *C_i_*)/2 under the diffusion-dominated condition and equals *C_p_* under the convection-dominated condition. *J_v_* is the fluid flow rate through the capillary wall, which is equivalent to the lymph flow rate, σ is the osmotic reflection coefficient for the restricted movement of molecules by convective flux, and *PS* is the permeability–surface area product. Equation (1) can be converted to Equation (2) by using the ratio of convection against diffusion, Peclet number (*Pe*):

(2) $$J_{t}=J_{v}\left(1-\sigma \right)C_{p}+PS\left(C_{p}-C_{i}\right)\frac{Pe}{\exp\left(Pe\right)-1}$$

(3) $$Pe=\frac{J_{v}\left(1-\sigma \right)}{PS}$$

Furthermore, many studies use a simpler equation which assume that the diffusive transport is negligible for IgG [97,107,119,125]:

(4) $$J_{t}=J_{v}\left(1-\sigma \right)C_{p}$$

The two-pore model expresses the net transport rate as follows [109]:

(5) $$J_{t}=J_{L}\left(1-\sigma_{L}\right)C_{p}+PS_{L}\left(C_{p}-\frac{C_{i}}{R}\right)\frac{Pe_{L}}{exp\left(Pe_{L}\right)-1}+J_{S}\left(1-\sigma_{S}\right)C_{p}+PS_{S}\left(C_{p}-\frac{C_{i}}{R}\right)\frac{Pe_{S_{}}}{exp\left(Pe_{S}\right)-1}$$

where *J_L_* and *J_S_*, *Pe_L_* and *Pe_S_*, and *σ_L_* and *σ_S_* are the fluid flow rates, Peclet numbers, and the osmotic reflection coefficients through the large and small pores of the capillary membrane, respectively. *R* is the partition coefficient of mAbs between the vascular and extravascular space, usually set at 1.

*J_L_* and *J_S_* are described by the following equation:

(6) $$J_{L}=J_{iso}+\alpha_{L}L$$

(7) $$J_{S}=-J_{iso}+\alpha_{S}L$$

where *J_iso_* is the recirculation flow rate, *L* is the lymph flow rate, and α*_L_* and α*_S_* are the hydraulic conductivities through the large and small pores, respectively.

Some studies adopted the one-pore model as a less parameterized model for expressing the extravasation of mAbs [31,97,107,110,117,118,119,126]. The two-pore model was also used in some studies [106,108,109,113,114,115,116,127]. As mentioned by Rippe and Haraldsson [128], despite the overparameterization, the two-pore model improves the overestimation of diffusive transport compared with the one-pore model. On the other hand, studies using a simplified one-pore model omitting diffusion also showed good prediction of mAb PK [97,107,110,117,118,119,126]. When applying the PBPK model to molecules that are smaller than mAbs, the two-pore model might be suitable because the diffusive transport may strongly contribute to extravasation of smaller-sized molecules and more accurate estimation of diffusive transport is required [113,114]. However, the recirculation flow rate and the osmotic reflection coefficients used in the two-pore model are different across the literature; there is at most a 391,500-fold difference in the recirculation flow rate of spleen (2.0 × 10^−7^ to 0.08 mL/min/g tissue) and at most a 5-fold difference in the lymphatic reflection coefficient [108,109,115,116,128].

4. Volumes of tissues and sub-tissue compartments

Although studies used comparable tissue volumes and interstitial volumes, the volumes of the endosomal and vascular compartments varied greatly between studies: there was around a 20- and 50-fold difference in endosomal and vascular volumes [33,106,107,108,109,110,111,113,114,115,116,117,118,119,120,121,122,123,128,129,130].

Because of the wide variability between reports in estimated parameters, the predicted mAbs concentrations in each tissue compartment might also vary widely depending on the parameters used. A robust model that can be applied to a variety of mAbs and species should be established by compiling experimental data.

In contrast to the complexity and variability of parameters in the full PBPK models, the minimal PBPK models have a simpler structure with a limited number of organs and can be used to describe the concentration profiles in specific tissues [112,121,122,123,129,131,132,133,134,135,136]. With minimal PBPK models, it is easy to incorporate additional tissue compartments, such as tumor and skin as the site of absorption, and additional kinetics, such as target antigen binding into the specific tissue compartments.

### 4.2. Use of PBPK Model to Mechanistically Describe and Predict mAb PK and PD

Using full or minimal PBPK models, many studies have characterized the mechanisms underlying the kinetics and tissue distribution of mAbs [97,107,108,110,119,120,121]. Some reports integrated the FcRn-mediated recycling process into their full PBPK model with estimating unknown parameters, such as FcRn concentration, in each tissue [107,108]. Based on these estimated parameters, some reports investigated the accuracy in predicting mAb pharmacokinetics in the presence of high and low concentrations of endogenous IgG in WT and FcRn-KO mice [119] and investigated the effect of the interplay of binding affinities against the target and FcRn and/or pH dependency in FcRn-binding properties on mAb pharmacokinetic output [97,110,121]. As for predicting the effect of in vitro FcRn-binding affinity on the half-life of mAbs, the catenary PBPK model [110,120] showed better accuracy than the equilibrium model.

There have been several reports constructing full [111,130] and minimal [122,137] PBPK models to characterize the mechanism of absorption after subcutaneous administration. In this process, mAbs are directly injected into the interstitial space and then absorbed into the vascular space through convective lymphatic transport or diffusive transport across the vascular endothelial space. Gill et al. proposed the two-pore transcapillary transport model to express subcutaneous absorption [130]. Their model successfully explained the plasma concentration profiles of various-sized therapeutic proteins in the range of 8 to 150 kDa after subcutaneous administration. They also mentioned that the diffusion process did not fully account for mAb distribution into the systemic circulation after subcutaneous dosing. The exact parameters related to the flow of fluid through the lymphatic vessels are unknown. Hu and D’Argenio included the processes of pre-systemic degradation, trafficking, and absorption of mAbs in the subcutaneous injection site [111]. They revealed that the lymphatic uptake pathway accounts for 91.6 to 99.0% of the total subcutaneous absorption, which is consistent with a previous report [138]. Zhao et al. used the minimal PBPK model with the one-pore capillary transport to also show the large contribution of lymphatic flow to T_max_ and the bioavailability of mAbs after subcutaneous administration [122]. Varkhede and Forrest showed that the pI of mAbs affects subcutaneous bioavailability by applying the minimal PBPK model to the plasma concentration data of several mAbs in humans [137].

The TMDD process should be included in the PBPK model to explain the nonlinear pharmacokinetics caused by target binding and internalization. Multiple parameters are needed to express the TMDD: the expression levels, tissue distribution, and turnover rate of target antigens, and the internalization rate of mAb–target antigen complex. Abuqayyas et al. included the TMDD for two mAbs against tumor-specific carcinoembryonic antigen (CEA) in two xenograft tumors by using the measured CEA concentrations in excised tumors or tumor cells, the internalization rate of the mAb–target antigen complex measured in an in vitro study, and the KD measured by SPR or cell binding assays [117]. The model accurately predicted the impact of these parameters on mAb pharmacokinetics, suggesting the predictability of human pharmacokinetics and tissue disposition using the PBPK model approach. Glassman et al. incorporated TMDD into all tissue compartments for the cynomolgus monkey and human PBPK model to describe the tissue distribution of several mAbs against several membrane targets by applying the IHC scores of target antigens to set their concentrations in each tissue [120,139]. The model successfully predicted the tissue distribution of some mAbs, further indicating the usefulness of the PBPK model in predicting tissue concentrations of mAbs and the receptor occupancies by knowing the target antigen expression patterns and turnover rates. This PBPK-model-based method can also be modified to predict pharmacodynamics and/or toxicity for the estimation of efficacious dosing regimen of mAbs during earlier periods of drug development.

The Simcyp consortium group provided the perspective on the quantification and verification of PBPK models for regulatory submission [140] based on the recently increasing applications of PBPK-PD modeling in clinical studies. There have been several reports combining a full or minimal PBPK model and PD model to explain the PKPD relationship for candidate selection and clinical prediction [122,123,129,131,136,141]. As reported by these studies, the optimal physicochemical profile of mAbs and the physiological characteristics of target patient populations for ideal efficacy can be determined by the PBPK-PD model which captures well the relationship between pharmacokinetics and efficacy. However, as mentioned above, the parameters used in each model were different, and this might affect the extrapolation of PKPD output. Although the quality of the PBPK model must be assessed, especially for clinical simulations for regulatory submissions, it is still difficult to fully validate the physicochemical and physiological parameters due to the lack of a sufficient in vivo data set. While the full PBPK model can predict the mAbs concentration in various tissues, the minimal PBPK model is also useful for more complicated simulations, including pharmacodynamics simulations.

## 5. Prediction of Human PKPD of ADC Using M&S

**Figure 4:**
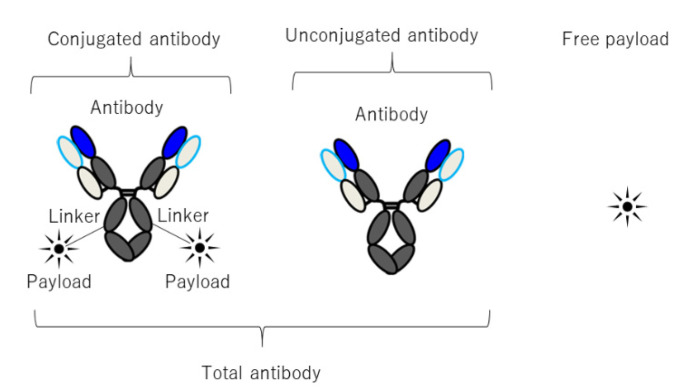
Structure and nomenclature of ADC.

ADC is a unique therapeutic mAbs modality that is mostly used for anticancer drugs. ADC consists of a mAb that binds to the target antigen, a payload with tumor-cell-killing activity, and a linker which combines the payload and mAb (Figure 4). The number of conjugated payload molecules per mAb is called the DAR (drug to antibody ratio). After administration, ADC binds to a target tumor antigen and enters the cell through target-antigen-mediated internalization. Then, the conjugated payload is released by the degradation of the mAb and/or the linker in the tumor cell lysosome. Tumor-cell-killing activity is exerted by the released payload. Antibody-dependent cellular cytotoxicity (ADCC)/antibody-dependent cellular phagocytosis (ADCP) and neutralization of antigen function also contributes to efficacy in some ADCs.

Pharmacokinetic aspects of ADC resemble those of mAb rather than its payload. That is, ADC shows a prolonged plasma half-life depending on the FcRn-mediated recycling. Nonlinear pharmacokinetics by TMDD is sometimes seen [142,143]. ADC also shows extravasation-limited distribution into tumor interstitial space [144]. However, due to the conjugation of the payloads, the PKPD of ADC has peculiar aspects. Namely, (1) the molecule is non-homogenous even if a site-specific payload conjugation technology is employed. (2) Cleavage of the linker during the systemic circulation yields a variety of molecules having different DAR [145]. (3) The complex payload release process in tumor including the bystander effect [146] makes it harder for PD modeling. (4) Each payload shows distinct PKPD profile that includes drug–drug interaction (DDI) [147,148]. With these complexities, modeling ADC PKPD has peculiar challenges while being an indispensable tool for ADC drug discovery and development.

### 5.1. PKPD Modeling of ADC

Although the linker is designed to be stable in plasma, ADC undergoes a variety of metabolic changes, including linker cleavage, after the administration. Therefore, heterogeneity of the molecule which results from the uneven release of the conjugated payload is observed. The quantification of distinct DAR is possible with LC–MS measurement, so ADC PK models reflecting the time evolution of individual DAR species were developed [149,150]. However, due to the complexity of LC–MS measurement, ELISA is often used as a simple alternative to assess the time evolution in DAR [151]. As a result, PK models describing the total mAb, conjugated mAb, and free payloads are often used [152,153]. The time evolution of such molecular species in plasma is described using a modified compartment model with molecular species conversion by the linker cleavage. The simplest way to calibrate these model parameters is to adjust the model parameter to recapitulate the in vivo plasma concentrations of total mAb and conjugated mAb. Sometimes, in vitro linker cleavage is measured to assess the cleavage kinetics [154]. Since PD/toxicodynamics (TD) of ADC can take place in a particular organ/tissue within a body, the PBPK model of ADC is also used for the characterization of organ/tissue-specific PD/TD effect. Because ADC has pharmacokinetic characteristics of both mAbs and small molecule drugs, the ADC PBPK model is often a combination of a mAb and small molecule PBPK model. For example, Shah et al. constructed a conjugated ADC PBPK model with five compartments (i.e., blood cell, plasma space, endosomal space, interstitial space, cell membrane, and cellular space). The distribution of the free payload is described by the tissue:plasma partition coefficient (K_p_) value estimated based on the physicochemical property of the payload [155,156]. Nonlinear pharmacokinetics of ADC caused by TMDD is described by the generic TMDD model which is frequently used in mAbs [157].

The pharmacodynamics of ADC has been described either with empirical or mechanistic models. For example, Jumbe et al. described the pharmacodynamics of trasutuzumab-DM1 (T-DM1) with a transit compartment model [158]. The model assumed that conjugated-antibody-dependent tumor cells transition to an inactive state and then transition to a dead state. During this process, other tumor cells proliferate. Based on this model, the TSC of conjugated ADC could be derived assuming no net tumor cell increase by the balance between the tumor cell death and tumor cell proliferation. TSC could be used for the in vitro–in vivo correlation (IVIVC) and as a reference for the dosing strategy. On the other hand, a more mechanistic description of pharmacodynamics is sometimes needed to gain insights for the drug discovery and development strategy. For that purpose, the Krogh cylinder model of tumor disposition is often used [159]. With this model, Cilliers et al. showed that the co-administration of trastuzumab and T-DM1 results in increased tumor penetration of T-DM1 by the masking of tumor peripheral antigen with trastuzumab, hence the enhanced antitumor activity [144]. Sigh et al. also used the Krogh cylinder model and showed that the more frequent dosing of trastuzumab-vcMMAE is preferred if the bystander effect is considered [160]. This is due to the prolonged and sufficient exposure of the free payload to antigen-negative cells in cases with a frequent dosing regimen.

### 5.2. Translational PKPD Prediction and Clinical Model Analysis of ADC

After the construction of a preclinical PKPD model, it must be extrapolated to humans ahead of clinical development. The estimation of the first-in-human (FIH) dose is especially important. For mAbs and small molecule drugs, several methods for human projection have been proposed. Chunze et al. performed a two-compartment-model analysis of 11 ADCs and tried several conventional extrapolation methods [161]. As a result, they concluded that the best extrapolation method for humans is single species allometry using pharmacokinetic data in cynomolgus monkeys with exponent = 1.

The population pharmacokinetic (popPK) model is used to determine the group average and covariates of pharmacokinetic parameters. Since the quantification of each molecular species with different DAR in clinic is difficult, a simpler model than that used in the preclinical stage is needed. One or two modeled molecular species are often selected from total mAb, conjugated mAb, conjugated payload, or free payload. Modeling approaches for currently approved ADCs in clinical studies are summarized in Table 1. As the covariates of popPK model, BW (body weight) and BSA (body surface area) were frequently identified and the insights were used for a dose-capping plan for enfortumab vedotin and brentuximab vedotin [162].

PopPK analysis also showed enhanced clearance of conjugated mAb of brentuximab vedotin [163] and T-DM1 [164] within liver dysfunction patients, and enhanced clearance of brentuximab vedotin in renal dysfunction patients [163]. In the meantime, the free MMAE clearance of brentuximab vedotin decreased in liver and renal dysfunction patients [163] and reduction in free MMAE clearance of polatuzumab vedotin was also observed in liver dysfunction patients [165]. As for brentuximab vedotin, the label recommends avoiding use in liver dysfunction patients and renal dysfunction patients were implemented based on these findings. DDI risk assessments were also performed using established popPK models coupled with in vitro and in vivo assay results. Currently, no particular DDI risk as a perpetrator of registered ADCs has been found. This is presumably due to the relatively low exposure of toxic molecular species under clinical dosing regimen, although some DDI risk as a victim drug remains.

Risk/benefit assessment in a clinical study and PII/PIII dose adjustment is often performed using the exposure–response model (ER model). For example, ER model analysis of gemtuzumab ozogamicin, an ADC which was once registered but withdrawn due to hepatoxicity, revealed that C_max_ contributes to the toxicity while efficacy is independent of C_max_. Based on the analysis, fractionated dosing of 3 mg/m^2^ was implemented instead of 9 mg/m^2^, leading to the later re-approval of gemtuzumab ozogamicin [166,167]. One notable point for ER analysis in ADC is that the correlation of particular analyte exposure to the PD/TD depends on the ADC molecule in question.

Based on the recent success of ADCs, many are now under development. Given the complexity of the MoA and structural format, model-based drug discovery and development would be inevitable for this particular modality. In this section, we summarized the current methodology and findings in ADC model analysis. Despite a substantial effort from preclinical to clinical, the estimation of the therapeutic index (TI) of ADC has not been so successful. This has led to the failure of some clinical trials. Considering the relatively successful modeling of the systemic PK of the ADCs, a solution might be found within PD modeling, especially of ADC tumor distribution. The Krogh cylinder model is currently the best at describing ADC distribution in tumor interstitial space. Some factors related to the PD are implicated by the sensitivity analysis of the Krogh cylinder model in preclinical models. Another important field for ADC modeling should be immuno-oncology therapy. There is ample evidence of immune cell activation by ADC through the Fc-mediated process. In addition, the evidence of immunogenic cell death induction by payloads has been accumulated, but both mechanisms have unfortunately not yet been fully modeled. Further efforts are needed for the modeling and quantitative prediction of ADC in immuno-oncology. Although challenged with these difficulties, PKPD models of ADC are all the more needed for the quantitative understanding of ADC, which is hard to capture by any other methods.

## 6. QSP-Model-Based Prediction of Human PKPD

QSP is a research area that aims to model the mechanistic processes found in complex biology and link them to the PKPD of drugs. Previously, traditional model-based approaches and relatively simple model approaches such as TMDD had been the primary choices for human PKPD prediction. However, due to severe competition in drug discovery and development and deep understanding of biology, several researchers have tried more complex and mechanistic M&S approaches such as QSP modeling. QSP modeling has recently been applied to problems such as target antigen selection, biomarker identification, and the prediction of clinical pharmacodynamics [179]. Although QSP modeling is still an immature methodology in drug discovery and development, it will become a major tool for predicting the human PKPD of new drug candidates in the near future.

### 6.1. QSP-Model-Based Prediction of Pharmacodynamics for Masked Tumor-Activated Antibody

As described earlier, mAbs have a high specificity to the target antigen, resulting in a low risk of off-target toxicity. However, if the target antigen expresses on both the target cell and normal cell, it is difficult to control on-target toxicity with maintaining desirable efficacy. PROBODY therapeutics (Pb-Tx) has been developed as a prodrug antibody which can be activated by tumor-specific proteases. Once the protease-cleavable domain is cleaved by tumor-specific proteases, the mask domain can be released; then, Pb-Tx binds to the target antigen in the tumor. Due to the complex and multi-step activation process of Pb-Tx, the QSP model was developed and translational PKPD prediction of Pd-Tx was conducted.

Stroh et al. first reported the construction of the QSP model for Pb-Tx using anti-CD166 Pd-Tx [180]. Five different anti-CD166 Pd-Tx with different protease-cleavable domains and mask domains and parent anti-CD166 antibodies were evaluated in cynomolgus monkeys for model construction and parameter estimation. The obtained results were captured by the QSP model by fitting each parameter; then, the parameters were scaled from cynomolgus monkeys to humans. Since the QSP model in cynomolgus monkeys did not have tumor compartment, tumor compartment and related parameters were added in the human QSP model based on information from the literature. The estimated human QSP model was used to select the optimal mask domain property and predict efficacious dosage in clinical situations. Stroh et al. further expanded their QSP model to anti-PD-L1 Pd-Tx, CX-072 [181]. The anti-CD166 Pd-Tx-based QSP model was modified for anti-PD-L1 Pd-Tx based on preclinical pharmacokinetic data of CX-072 in cynomolgus monkeys and clinical pharmacokinetic data of atezolizumab which is a conventional anti-PD-L1 antibody. The modified QSP model well predicted the pharmacokinetics of plasma total CX-072 and masked CX-072 concentration in the clinical study. Moreover, the observed intratumor PD-L1 occupancy which was calculated based on the observed intratumor activated CX-072 concentration in humans was successfully predicted by the QSP-model-based approach. This analysis supported the design of effective dosing regimen of CX-072 in clinical settings.

### 6.2. QSP-Model-Based Prediction of Pharmacodynamics for Bispecific T Cell Engager

Bispecific T cell engager (TCE) is a bispecific antibody targeting CD3 on T cells and tumor antigens on tumor cells, bringing them in close proximity and inducing T-cell-dependent tumor-killing activity [182]. Although several TCEs are being developed in clinical trials, there is a lack of translational strategy of PKPD due to complex MoA. Thus, the QSP model which can describe a complex biology system and disease condition/progression has been applied to perform mechanism-based translation of preclinical PKPD of TCEs to humans. TCEs form TCE-T cell–tumor cell trimer and show T-cell-dependent tumor-killing activity. Therefore, the mechanistic incorporation of trimer formation in the tumor microenvironment in the QSP model can be a key factor to consider the pharmacodynamics of TCEs.

**Figure 5:**
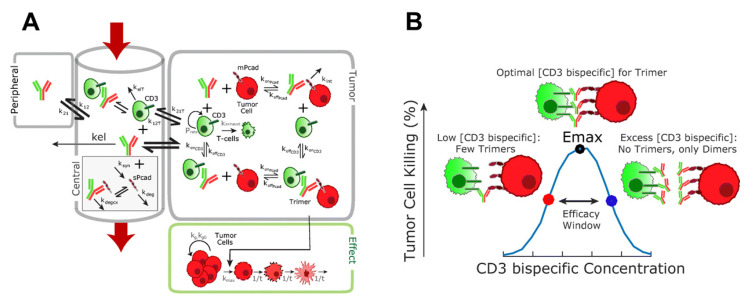
(**A**). QSP model for PF-06671008, bispecific anti-CD3/P-cadherin antibody. (**B**). Schematic of the bell-shaped tumor-cell-killing activity based on the concentration of trimer. Figure was reproduced from [184].

Campagne et al. applied the QSP model to quantitatively describe PKPD of flotetuzumab, a bispecific anti-CD3/CD123 antibody, in cynomolgus monkeys [183]. In cynomolgus monkeys, T cell and CD123-positive cell dynamics after flotetuzumab injection were monitored as pharmacodynamic parameters. Obtained pharmacokinetics and pharmacodynamics were analyzed by the QSP model and trimer (flotetuzumab-T cell-CD123 positive cell) concentration was predicted. Although the constructed model was not scaled to humans in this report, model structure and estimated parameters can be translated from cynomolgus monkeys to humans using the appropriate scaling approach. Betts et al. developed a QSP model for PF-06671008, bispecific anti-CD3/P-cadherin antibody, and predicted pharmacodynamics in humans based on preclinical data [184]. First, the QSP model (Figure 5A) was constructed from results of mouse xenograft efficacy study data by incorporating several mechanisms such as pharmacokinetics of PF-06671008, CD3/P-cadherin expression on T cells and tumor cells, T-cell dynamics in blood and tumor, and the formation of PF-06671008-T cell–tumor cell trimer. Trimer concentration in tumor was assumed to determine tumor-killing activity in this model (Figure 5B). Then, the constructed model and estimated parameters were translated from mouse to human. The final human QSP model predicted trimer formation in tumor in clinical situations and it was found that P-cadherin expression level in tumor cells and T-cell number in tumor were highly sensitive to trimer formation in sensitivity analysis. This analysis provides framework of QSP-model-based prediction of human PKPD of TCE and importance of each parameter on trimer formation. Hosseini et al. developed a QSP model to translate pharmacodynamics of a bispecific anti-CD3/CD20 antibody, mosunetuzumab, from cynomolgus monkeys to humans [185]. Plasma and tissue T cell/B cell kinetics and cytokine kinetics after mosunetuzumab injection were evaluated in cynomolgus monkeys and captured by the QSP model. Then, estimated parameters in cynomolgus monkeys were translated to humans. Tumor compartment was additionally included in the human QSP model. When the human QSP model was constructed in this report, clinical data of blinatumomab, which is an approved bispecific anti-CD3/CD19 antibody fragment, was utilized to optimize parameters. Since blinatumomab has similar MoA with mosunetuzumab, utilizing clinical data of blinatumomab could be a valuable approach to improve the predictability. The constructed human QSP model predicted T-cell activation, cytokine kinetics, and anti-tumor efficacy in humans and contributed to optimized clinical efficacious dosing strategy with migrating cytokine release. Recently, a similar approach without clinical blinatumomab data was reported in another bispecific anti-CD3/CD20 antibody, glofitamab [186].

Although above reports predicted human pharmacodynamics based on in vivo data, Chen et al. reported an in vitro-data-based pharmacodynamics prediction approach for a bispecific anti-CD3/P-cadherin antibody [187]. First, an in vitro QSP model was constructed using in vitro cytotoxicity kinetic data and binding affinity data. Trimer (TCB-T cell-tumor cell) concentration in the constructed model was assumed to determine tumor-killing activity. Then, estimated in vitro QSP model parameters were scaled to human in vivo parameters. T cell and tumor cell number, and P-cadherin expression in tumor cell and CD3 expression in T cell were replaced by human in vivo parameters. Other parameters were assumed to be the same with the in vitro QSP model. Pharmacokinetics in humans was predicted from cynomolgus monkeys by the allometric scaling approach. The final QSP model predicted trimer formation in human tumors and contributed to the first-in-human dose design. Song et al. also reported a similar in vitro-data-based pharmacodynamics prediction approach for a bispecific anti-CD3/EpCAM antibody, M701, to treat malignant ascites [42]. In vitro-data-based quantitative QSP modeling predicted the ascites exposure–cytotoxicity relationship of M701. In vitro experiments can be flexibly conducted in several conditions by changing each factor such as T cell and tumor cell number, source of T cell and tumor cell, and TCB concentration. Thus, this approach can construct a QSP model more logically based on actual experiment data. The optimal combination of in vivo and in vitro data could be important to improve model construction, parameter estimation and human prediction accuracy.

### 6.3. Future Perspectives for QSP-Model-Based Prediction of Pharmacodynamics

QSP-model-based pharmacodynamics prediction of mAbs in humans becomes an important approach especially when a MoA is complex and the translation from preclinical data to humans is not simple. Although major reports on QSP modeling for mAbs have focused on cancer, its application to other diseases, such as Alzheimer’s disease [188], COVID-19 [189], and asthma [190], has recently been reported. Furthermore, for an integrated understanding of complex disease biology, disease-based QSP model platforms have also been developed for several diseases such as heart failure [191], inflammatory bowel disease [192], Parkinson’s disease [193], and type 2 diabetes mellitus [194]. These disease-based QSP model platforms can be applied in any therapeutic modality, including mAbs. Therefore, the application of QSP modeling for the discovery and development of mAbs will be expanded in several diseases. Since the QSP model incorporates numerous numbers of parameters, the reliability of each parameter can be an important factor for predictability. Thus, the accurate estimation of each parameter becomes crucial for QSP modeling. The accumulation of scientific evidence to estimate parameters will increase the reliability and enable QSP modeling to be a more familiar tool for human PKPD prediction in the near future.

## 7. Conclusions

As we reviewed, there has been extensive progress in M&S-based quantitative approaches for predicting the PKPD of mAbs in humans. Since the MoAs of current clinical candidate molecules are often complex, mechanistic models have been frequently used. On the other hand, although the recent trend in M&S has been toward more complex mathematical models, an optimal model should be selected based on the purpose of the study and the quality of available data sets. Additionally, the quality of data sets can be an important factor for M&S analysis. Therefore, several points such as study design, bioanalysis method, and immunogenicity should be carefully considered to analyze the data by M&S. Due to intense competition in the discovery and development of mAbs, the increase in clinical probability can be a powerful advantage in the pharmaceutical industry. Thus, it is essential to accurately predict human PKPD before starting a clinical trial. Additionally, because M&S requires mathematical and computational technologies, progress in big data analysis, artificial intelligence (e.g., machine learning (ML)), and the development of analytical software will contribute to future advancement of M&S. Recently, ML-based PKPD prediction has been gaining interest. Indeed, ML-based popPK analysis shows several successful examples such as T-DM1 [195]. However, application of such a method to translational research of antibody therapeutics is now scarce. This is probably due to the scarcity of the preclinical data, which is not sufficient for the learning process of the model parameters. Further research needs to be performed in the application of ML-based human projection. The integration of multiple datasets and modeling strategies is needed to maximize the value of M&S. The pharmaceutical industry must rightly understand and utilize M&S in order to improve the probability of success of new drug candidates.

## Figures and Tables

**Table 1:** Modeling approaches of approved ADCs in clinical development.

**ADC Name**	**Approval** **Year**	**Target**	**Payload**	**Modeled Analytes** **in popPK Model**	**Model Structure** **and Description**
Gemtuzumab ozogamicin	2017;2000	CD33	Calicheamicin	tAb	2-COMP, LE + TDE
				Unconjugated Calicheamicin	2-COMP,1stF, LE
Brentuximab vedotin	2011	CD30	MMAE	ADC	3-COMP, LE
				Unconjugated MMAE	2-COMP, TDF, LE
Ado-trastuzumab emtansine	2013	HER2	DM1	ADC	2-COMP, LE
Inotuzumab ozogamicin	2017	CD22	Calicheamicin	ADC	2-COMP, LE + TDE
Moxetumomab pasudotox	2018	CD22	Pseudomonas exotoxin A	ADC	1-COMP, CDLE
Polatuzumab vedotin	2019	CD79	MMAE	Conjugated MMAE	2-COMP, LE + TDE + MME
				Unconjugated MMAE	2-COMP, LF + NLF, LE + MME
Enfortumab vedotin	2019	Nectin-4	MMAE	ADC	3-COMP, LE
				Unconjugated MMAE	2-COMP, LE
Fam-trastuzumab deruxtecan-nxki	2019	HER2	DXd	ADC	2-COMP, LE
				Unconjugated DXd	1-COMP, 1stF, LE
Sacituzumab govitecan	2020	Trop-2	SN-38	Conjugated SN-38	1-COMP, LE
				Unconjugated SN-38	2-COMP, 1stF, LE
Loncastuximab tesirine	2021	CD19	PBD	tAb	2-COMP, LE + TDE
				ADC	2-COMP, LE + TDE
Tisotumab vedotin	2021	Tissue factor	MMAE	ADC	2-COMP, LE + MME
				Unconjugated MMAE	1-COMP, LE
**ADC Name**	**Analysis of** **Liver Dysfunction** **Patients**	**Analysis of** **Renal Dysfunction** **Patients**	**Payload DDI Risk** **(Perpetrator)** **Assessment Approach**	**Analytes for** **ER Model**	**Ref.**
Gemtuzumab ozogamicin	popPK: NCI criteria	popPK: CrCL	In vitro effective concentration vs. Cp	tAb	[168]
Brentuximab vedotin	Clinical study: Child-Pugh	Clinical study:CrCL	In vitro effective concentration vs. Cp and clinical study	ADC, MMAE	[169]
Ado-trastuzumab emtansine	Clinical study: Child-Pugh	popPK: CrCL	In vitro effective concentration vs. Cp	ADC, tAb, DM1	[170]
Inotuzumab ozogamicin	popPK: NCI criteria	popPK: CrCL	In vitro effective concentration vs. Cp	ADC	[171]
Moxetumomab pasudotox	popPK: NCI criteria	popPK: CrCL	NA	ADC	[172]
Polatuzumab vedotin	popPK: NCI criteria	popPK: CrCL	In vitro effective concentration vs. Cp and PBPK model	MMAE, Conjugated MMAE, tAb	[173]
Enfortumab vedotin	popPK: NCI criteria	popPK: CrCL and Clinical study: CrCL	In vitro effective concentration vs. Cp	ADC, MMAE	[174]
Fam-trastuzumab deruxtecan-nxki	popPK: NCI criteria	popPK: CrCL	In vitro effective concentration vs. Cp and clinical study	ADC, DXd	[175]
Sacituzumab govitecan	popPK: NCI criteria	NA	NA	IgG, total-SN-38, free-SG-38, SN-38G	[176]
Loncastuximab tesirine	popPK: NCI criteria	popPK: CrCL	In vitro effective concentration vs. Cp	ADC	[177]
Tisotumab vedotin	popPK: NCI criteria	popPK: CrCL	No dedicated study (reference to brentuximab vedotin)	ADC, MMAE	[178]

1-COMP: 1 compartment model, 2-COMP: 2 compartment model, 3-COMP: 3 compartment model, LF: linear formation, NLF: nonlinear formation, 1stF: 1st order formation, LE: linear elimination, TDE: time dependent elimination, MME: Michaelis–Menten elimination, CDLE: cycle-dependent linear elimination, NCI criteria: National Cancer Institute classification system criteria, CrCL: creatinine clearance, ADC; conjugated antibody, tAb: total antibody, IgG: unconjugated antibody.

## Data Availability

Data is contained within the article.
